# NIR Excitation in Atomically Precise Nanoclusters via Two-Photon and Three-Photon Absorption

**DOI:** 10.1021/acs.accounts.5c00832

**Published:** 2026-03-19

**Authors:** Agata Hajda, Patryk Obstarczyk, Joanna Olesiak-Bańska

**Affiliations:** Institute of Advanced Materials, Wroclaw University of Science and Technology, Wybrzeze Wyspianskiego 27, 50-370 Wrocław, Poland

## Abstract

Gold nanoclusters exhibit unique optical properties, with absorption similar to that of molecular systems, ranging from the UV to near-infrared (NIR) region with tunable photoluminescence. Nanoclusters also present outstanding multiphoton properties, with the possibility of excitation in the NIR-I and NIR-II ranges via two- or three-photon absorption. Multiphoton microscopy, which relies on multiphoton excitation, offers additional advantages such as the selective excitation of molecules only at the focal point, enabling true 3D optical sectioning and reducing photobleaching and phototoxicity compared with one-photon microscopy. Our review organizes the current knowledge on the multiphoton absorption of noble metal nanoclusters, highlighting key recent advances in the field and addressing current challenges, with particular emphasis on the potential of these nanostructures in biological NIR imaging applications. The Account starts with an introduction to the basis of multiphoton absorption and multiphoton microscopy advantages, followed by examples of applications of nanoclusters as two- and three-photon absorbers and NIR emitters. Thus, current trends and further perspectives clearly show that the application of multiphoton excitation and noble metal nanoclusters provides a highly beneficial approach for efficient NIR bioimaging.

## Graphical abstract

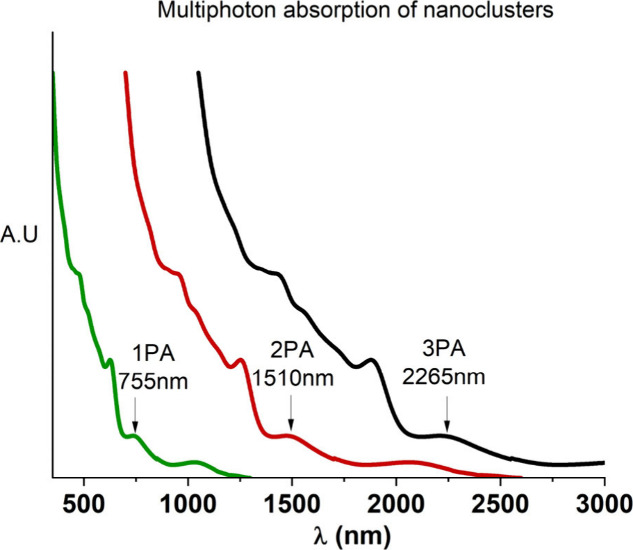

## Key References

-

Obstarczyk, P. ; Kazan, R. ; Bürgi, T. ; Samoć, M. ; Olesiak-Bańska, J. Two-Photon and Three-Photon Circular Dichroism of Au38 Gold Nanoclusters Enantiomers. J. Am. Chem. Soc. 2024, 146­(51), 35011–35015 10.1021/jacs.4c12321 39656153 PMC11931527).^89^
-

Hajda, A. ; Guha, R. ; Copp, S. M. ; Olesiak-Bańska, J. Two-Photon Brightness of NIR-Emitting, Atomically Precise DNA-Stabilized Silver Nanoclusters. Chem. Sci. 2025, 16­(4), 1737–1745 10.1039/D4SC05853D 39720144 PMC11664824).^98^
-

Pniakowska, A. ; Kumaranchira Ramankutty, K. ; Obstarczyk, P. ; Perić Bakulić, M. ; Sanader Maršić, Ž. ; Bonačić-Koutecký, V. ; Bürgi, T. ; Olesiak-Bańska, J. Gold-Doping Effect on Two-Photon Absorption and Luminescence of Atomically Precise Silver Ligated Nanoclusters. Angewandte Chemie - International Edition 2022, 61­(43), e202209645 10.1002/anie.202209645 36005739 .^70^

## Introduction

Photoluminescence (PL) is a process of light emission triggered by the absorption of photons, usually from the ultraviolet (UV) and visible ranges. It is widely applied in the detection and imaging of biomolecules and analytes *in vitro* and *in vivo*. In terms of *in vivo* imaging, especially useful is imaging in NIR biological windows, ranges of wavelengths above 700 nm, where light absorption and scattering by tissues are the weakest.^1,2^ There are two biological windows: NIR-I (700–950 nm) and NIR-II (1000–1700 nm).^3^ In the literature, the short-wave infrared (SWIR) region^4^ is also defined between 1000 and 2000 nm. Excitation in biological windows translates to deeper light penetration into the sample and improved visualization of underlying structures with improved three-dimensional (3D) imaging.^5^ These benefits are even more pronounced for the NIR-II window; however, obtaining emission and/or excitation in this region is a challenging task. Here, change from one-photon excitation (1PE) to two-photon excitation (2PE) or three-photon excitation (3PE) may be an answer for efficient fluorophore excitation above 1000 nm.^6,7^ The process of two-photon absorption (2PA) or three-photon absorption (3PA) can be utilized in multiphoton microscopy, where fluorescence is induced by the simultaneous absorption of two or three photons, followed by light emission (Figure 1a,b). Additionally, in multiphoton microscopy, only molecules at the focal point are excited; thus, intrinsic 3D sectioning is possible (Figure 1d). However, for efficient signal and selectivity in multiphoton microscopy, suitable fluorescent probes are needed.

**Figure 1:**
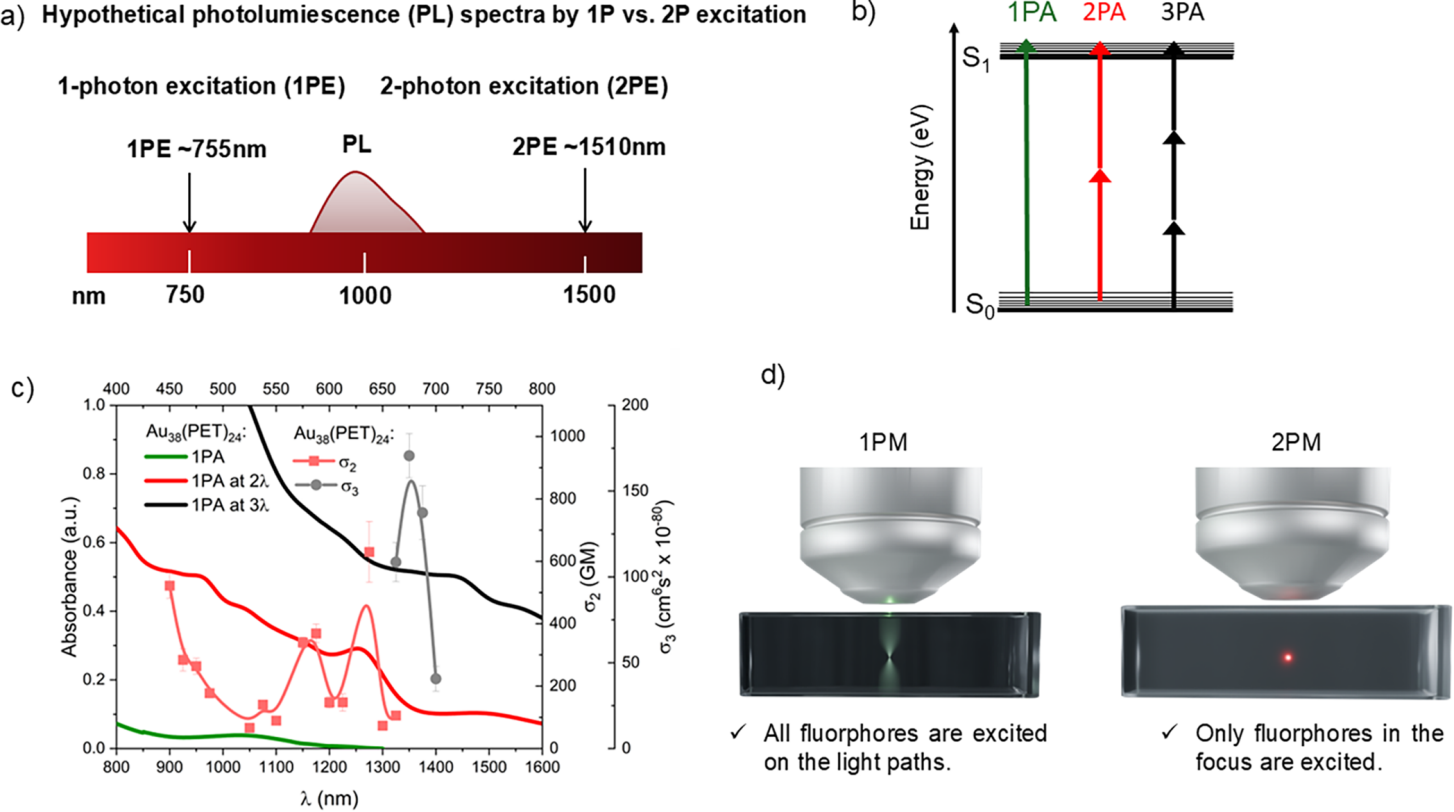
(a) Schematic illustration of wavelengths used in 1PE and 2PE and the obtained photoluminescence spectrum. In most cases, the PL spectrum is constant regardless of the type of excitation. (b) Simplified Jablonski diagram for 1PA, 2PA, and 3PA. (c) Experimentally obtained 2PA and 3PA cross sections of Au_38_(PET)_24_ (PET, phenylethyl mercaptan), marked with squares (σ_2_) and circles (σ_3_), respectively (B-spline lines were introduced to guide the eye). The 1PA spectrum is shown (green line) and replotted versus twice (red line) and three times (black line) the wavelength. Reproduced from ref 89. (d) Schematic illustration of the difference in 1PM and 2PM signal excitations.

A broad group of fluorophores consists of small organic compounds. However, rendering them suitable for multiphoton absorption in the NIR-II region is a challenging task. They suffer from multiple drawbacks: relatively low photostability (as compared to that of nanomaterials^8^) and poor water solubility due to the presence of aromatic rings and conjugated bonds, often limiting their use in biological environments. Due to the different selection rules for one-photon and multiphoton absorption, materials that are efficiently excited upon one-photon absorption (1PA) do not have to present strong 2PA or 3PA in a corresponding range of wavelengths. Organic compounds applied in two-photon bioimaging in the NIR-II region^7^ were mostly not optimized in terms of 2PA properties. 3PA is significantly less studied than 2PA, and a limited number of fluorophores were presented for three-photon microscopy (3PM).^9–11^ By shifting the emission toward red, yet another challenge appears: the energy gap between the ground state and the excited state decreases, which favors nonradiative relaxation processes and diminishes the photoluminescence quantum yield (PLQY). Thus, the number of fluorophores with strong 2P/3PA above 1000 nm and simultaneous efficient PL is very limited.

The ideal candidates here to address this gap are noble metal nanoclusters, which present strong two- and three-photon properties^12^ and high photostability and emission in the NIR-I^13^ and NIR-II regions.^14^ A brief review of the fundamental principles and measurement methods of multiphoton absorption is provided prior to discussing the specific optical properties of metal nanoclusters.

## Multiphoton Absorption and Photoluminescence

### Theoretical Background

Multiphoton-excited photoluminescence arises when fluorophores absorb two or more photons simultaneously (Figure 1b), enabling excitation in the NIR-I/II ranges under high photon fluxes (approximately 1 kW/cm^2^). For example, two-photon absorption (2PA) constitutes a third-order nonlinear process, and its efficiency can be quantified using two principal experimental approaches: (*i*) the Z-scan technique^12,15–18^ and (*ii*) the two-photon excited luminescence technique (2PEL)^19–22^ (Scheme 1).

**Scheme 1:**
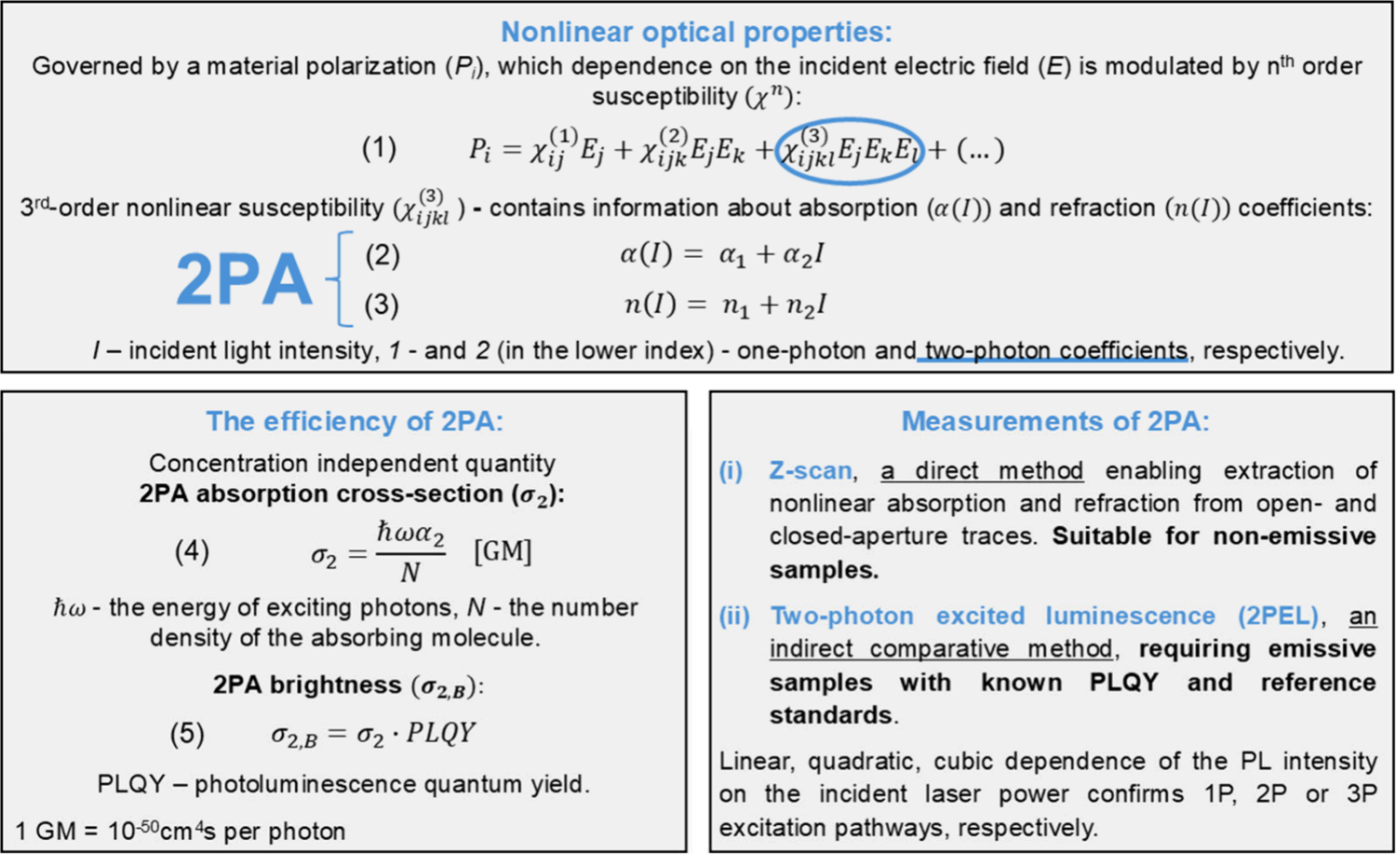
Overview of the Key Principles Underlying Nonlinear Optical Properties, Together with the Parametrization of Process Efficiency and the Principal Experimental Approaches Employed for Their Measurement

### Advantages of Multiphoton Excitation

Two-photon microscopy (2PM) is widely used in the imaging of complex, deep-lying structures like the brain^23^ and kidneys^24^ and also in intravital imaging^25,26^ and longitudinal imaging.^27^ In contrast to 1PE, 2PE operates at NIR wavelengths. NIR-I-to-NIR-I two-photon imaging (emission in NIR-I, excitation in NIR-I through 2PE) provides even 4 times deeper penetration into the tissues than imaging with probes presenting visible-range emissions.^28^ Even better results are obtained using NIR-I emission and NIR-II excitation, through 2PA. In recent years, this approach gave the largest penetration depths and the best spatial resolution of *in vivo* two-photon imaging up to 1.6–1.8 mm.^29,30^ Moreover, excitation via multiphoton absorption offers the greater excitation confinement, as the process occurs solely in the focus of the beam (Figure 1d). 3PM is mostly used in deep brain imaging,^31–33^ as the shift from 2PA to 3PA results in even stronger excitation confinement, which is crucial for high-contrast bioimaging. An overview of 2PM and 3PM in terms of bioimaging application is provided in ref 34.

High scattering and autofluorescence in the NIR-I range can be even further reduced when imaging (emission and excitation) operates at NIR-II wavelengths.^35,36^ In terms of one-photon NIR-II excitation followed by NIR-II fluorescence, there are examples in the literature that present an extremely high imaging depth (1.1 mm for 1P confocal microscopy)^37^ and even up to a few millimeter depth for other 1PM techniques.^38–41^ However, hardly any papers report NIR-II emission and excitation via 2PA in the NIR-II region^42–44^ due to limited access to NIR range detectors and a lack of suitable fluorescent probes. Caldorala et al. overcame this problem by proposing a novel array of superconducting nanowire single-photon detectors (SNSPDs) and used LZ-1105 dye as a probe.^44^ It resulted in an imaging depth of >1.1 mm in the *in vivo* mouse brain. In this context, fluorophores that emit in NIR-II and can be efficiently excited via 2PA/3PA may further exploit the benefits of NIR-II imaging. This gap may be filled with noble metal nanoclusters. Most metal nanoclusters exhibit emission in NIR-I, but there are representatives exhibiting NIR-II emission.^13,14,45,46^ Additionally, noble metal nanoclusters present high multiphoton absorption over a broad range of excitation wavelengths, which is needed for NIR-II multiphoton excitation.

## Metal Nanoclusters as Multiphoton Absorbers and NIR Emitters

Monolayer-protected, atomically precise metal nanoclusters refer to a group of nanomaterials described by [M_*n*_(XR)_*m*_]^*q*^, where M_*n*_ stands for the coinage metal number, XR_*m*_ is the number of protecting ligands, and *q* is the overall nanocluster charge. Nanoclusters are ultrasmall (<2 nm in diameter), and their structures are hierarchically organized with three components: (*i*) a metallic core, (*ii*) metal­(I)–ligand staplelike motifs, and (*iii*) a capping ligand shell.^47^ Metal nanoclusters can be an exciting alternative to other NIR-emitting fluorophores due to their high photostability (especially for gold nanoclusters), possible water-solubility thanks to proper stabilizing ligands, atomic precision, and theragnostic abilities.^48^ Originally, thiolate-stabilized gold^49^ nanoclusters emitting in NIR-I had low PLQY;^50–53^ however, extensive effort in the community resulted in understanding how to tune and increase their PLQY. Possible modifications to improve PLQY are changes in the ligand shell or metal core composition.^54^ Au_25_ is one of the most broadly studied atomically precise nanoclusters, with emission in both NIR-I and NIR-II windows, but the PLQY below 1%. However, the increase in the NIR-II emission of Au_25_ can be realized by doping with other metals like Cu and Zn,^55^ modifying the ligand shell (e.g., with cysteine, as compared to glutathione (SG)^55^), incorporating a single Au_25_ nanocluster into bovine serum albumin (BSA) protein,^56^ or attaching an additional ligand layer with PDA (2,6-pyridine­dicarboxaldehyde).^57^ From all of these approaches, the highest PLQY was obtained for PDA-Au_25_(SG)_18_ nanoclusters with a value of 3.26%.^57^ NIR-II-emitting gold nanoclusters have already been used in one-photon tumor imaging,^58^ acute kidney injury imaging,^59,60^ and gastrointestinal imaging,^61^ showing their broad application potential. Due to their sizes being below the glomerular filtration threshold^62,63^ (∼5.5 nm), they present effective renal clearance^55,63–66^ and low toxicity.^64–67^ Gold nanoclusters also present high photostability under one-photon excitation,^66,68^ up to a few hours, while dye ICG is photobleached under the same experimental condition in a few minutes. It should be noted that the photobleaching process may be different when using pulsed lasers, which are employed for multiphoton excitation.^69^ Regarding metal nanocluster photostability, the composition of metal atoms in the core plays the main role, which was shown for silver nanoclusters;^69,70^ however, there is a need for more systematic studies.

One of the subgroups of noble metal nanoclusters is the DNA-stabilized silver nanocluster (Ag_N_-DNAs). They differ in structure from the above-mentioned monolayer-protected metal nanoclusters. Ag_N_-DNAs are composed of up to 30 silver cations and atoms, which are templated by single-stranded DNA oligomers.^71^ They present tunable emissions ranging from the visible to NIR. The DNA oligomer sequence determines the interaction with silver atoms, which affects the size and shape of formed nanoclusters. As a result, optical properties of Ag_N_-DNAs are also highly DNA sequence-dependent. Due to a large-scale investigation of sequence-dependent optical properties of Ag_N_-DNAs also for the intended NIR-emission,^72,73^ the palette of Ag_N_-DNAs with NIR emission and simultaneously with high PLQY expanded significantly in recent years. Ag_N_-DNAs can also emit on the border between NIR-I and NIR-II,^74–78^ which enables their application in NIR-II imaging. The first reported Ag_N_-DNAs with emission peaks above 950 nm were discovered by high-throughput NIR screening technology.^76^ Recently, DNA_2_–[Ag_28_Cl_2_]^14+^ was reported, with PLQY = 12% at 960 nm.^74^ This cluster has a rodlike shape and measures over 2 nm in length. It is an important discovery in terms of NIR-II emission of metal nanoclusters since thiolate-stabilized gold metal nanoclusters with rod-like geometry also have NIR-II emission with extremely high PLQY (up to 50% in solution and 75% in film).^79–81^ Rod-like geometry due to the rigid structure seems to promote efficient NIR-II emission.

### Two- and Three-Photon Absorption in Noble Metal Nanoclusters

The above-mentioned examples of NIR-I and NIR-II imaging using nanoclusters are obtained via 1PE. However, noble metal nanoclusters are great candidates for NIR-to-NIR multiphoton imaging since they emit in NIR-I and NIR-II, have a broad range of absorption that makes it possible to excite them via multiphoton absorption in a large range of wavelengths, and present high σ_2_ values. Band positions inferred from one-photon spectra at double or triple wavelengths (see the TOC) provide only preliminary guidance. Due to the distinctive selection rules, actual 2PA and 3PA bands may not follow the intensities and positions of corresponding 1PA spectra, and the determination of 2PA and 3PA properties must be supported by quantitative experimental analysis, e.g., via a Z-scan or 2PEL technique (see exemplary experimental 2PA and 3PA spectra of Au_38_(PET)_24_ (PET – phenylethyl mercaptan) in Figure 1c). Rich absorption spectra of noble metal nanoclusters may lead to a resonant 2PE at 1PA wavelengths, which may result in the double-resonance-based enhancement of σ_2_.^82^ ^,^ ^83^ The presentation of full 2PA and 3PA spectra of nanoclusters remain scarce in the literature, where single-wavelength measurements are usually presented.^12^ Our group already contributed to the field by reporting the full 2PA spectrum and dispersion of the nonlinear refractive index of Au_25_(Capt)_18_ (Capt – captopril),^84^ with σ_2_ of up to 24000 GM (at 550 nm resonant excitation). Su et al.^85^ presented nonlinear absorption cross sections, refraction cross sections, and the inverse of the saturated absorption intensity for Au_25_(DDT)_18_ and Au_38_(DDT)_24_ nanoclusters (DDT - 1-dodecanethiol) in the 525–725 nm range. They showed that the Au_38_(DDT)_24_ cluster exhibits order of magnitude larger σ_2_ values in comparison to those of Au_25_(DDT)_18_, which suggests that increasing the size of a cluster might enhance its NLO properties. Similarly, Russier-Antoine et al.^86^ observed that the σ_2_ values of Ag_11_(SG)_7_, Ag_15_(SG)_11_, and Ag_31_(SG)_19_ increase with higher noble atom content and that σ_2_ values increase as the excitation wavelength shortens. Yousefalizadeh et al. determined the σ_2_ values for a range of Au_25_(SR)_18_, Au_18_(SR)_14_, and Ag_25_(SR)_18_ as well as Au_38_(SR)_24_ under 1025 nm excitation (SR – thiols). The highest reported σ_2_ value in their study was determined for Au_38_(PET)_24_ (39300 GM). Interestingly, they also showed the ligand influence on multiphoton excitation, as for Au_25_(SR)_18_ clusters σ_2_ values range from 4520 to 164 GM, for SR=Me_2_PhS (Me_2_PhS = dimethylbenzenethiol) and the SG ligand, respectively. Therefore, the nanocluster size and the ligands are crucial in the development of efficient NIR excitation via multiphoton absorption. Sakamoto et al.^87^ studied the nonlinear optical properties of Au_36_(NP)_24_, Au_36_(Ph)_24_, and Au_36_(Cy)_24_ in the 800–950 nm range (NP, Ph, and Cy – naphthalene thiol, triphenylphosphine, and cyclopentanethiol, respectively). The highest σ_2_ values were equal to 6000 GM (at 800 nm), 3000 GM (at 816 nm), and 2000 GM (at 863 nm) for NP, Ph, and Cy ligands, respectively. Based on the additional theoretical calculations, they proposed that high σ_2_ values can be achieved by control over the density of states of Au clusters via exploiting the orbital hybridization between a metal core and a molecular ligand, which may lead to a double resonance effect.^82^

The nonlinear optical properties can be further tuned and enhanced through the nanoclusters’ self-assembly. Our group showed that 2PA cross sections in the NIR wavelengths range may be enhanced with covalent linking of individual clusters into dimers or trimers.^83^ We showed that in the resonant excitation region σ_2_ is ∼4 and ∼8 times higher than σ_2_ of the Au_25_(PET)_18_ monomer (for a dimer and a trimer, respectively). In the off-resonance spectral region, the trimers exhibit 4 times higher σ_2_ values in comparison to those of the monomers while the dimers exhibit enhancement factors of ∼2. The enhancement was also strongly visible in other studies, in NCs optimized for 2PEL. By the choice of bulky counterions and a suitable solvent, a 30-fold increase in 2PEL was obtained for Au_15_ and Au_18_ NCs.^88^

Recently, the first reports on higher-order NLO properties of noble metal nanoclusters and nanocluster excitation via three-photon absorption were presented. Our group reported Au_38_(PET)_24_ 2PA and 3PA spectra in the 800–1400 nm wavelength range, where 2PA was registered below 1325 nm, and 3PA in the 1350–1400 nm range.^89^ The highest σ_2_ value was equal to 630 GM at 1275 nm, and the highest σ_3_ was equal to 1750 × 10^–80^ cm^6^ s^2^ at 1350 nm. Additionally, the solvent effect on PL and multiphoton properties of Au_9_Ag_6_(SPh*t*OMe)_4_(DPPOE)_3_Cl_3_ (SPhtOMe – 4-methoxythiophenol and DPPOE – bis­(2-diphenylphosphinophenyl) ether) was studied.^90^ Photoluminescence of Au_9_Ag_6_(SPhtOMe)_4_(DPPOE)_3_Cl_3_ was shown to be solvent sensitive under one-, two-, and three-photon excitation. The corresponding 3PA cross-section (σ_3_) for Au_9_Ag_6_ nanoclusters was equal to 3.28 × 10^–76^ cm^6^ s^2^ photon^–2^ at 2100 nm, while σ_2_ was equal to 1100 GM at 1300 nm.

### Tuning 2P Brightness and NIR Emission in Thiolate-Protected Nanoclusters

Simultaneous tuning of NIR emission and 2PA is scarcely studied in the literature on metal nanoclusters. To address this challenge, our group investigated the impact of gold doping on optical properties of silver nanoclusters (in the one- and two-photon regimes).^70^ We synthesized a series of Ag_25–*x*_Au_*x*_(DMBT)_18_ nanoclusters where *x* = 0, 1 and 5–10 (DMBT – 2,4-dimethylbenzenethiolate). All of the nanoclusters presented broad 1PA spectra (Figure 2a) and photoluminescence from the 870 to the NIR-II region (Figure 2b). Even low-intensity emission in the NIR-II region can be sufficient in applications in NIR-II imaging, as was already used for organic dyes. For example, indocyanine green (ICG), an FDA-approved dye, has its fluorescence maximum within the 800–860 nm range but the tail of emission above 1000 nm.^91,92^ ICG is a widely used probe in preclinical and clinical NIR-II imaging.^93,94^ Ag_25_(DMBT)_18_ had photoluminescence at around 950 nm, with a PLQY equal to 3.08%. However, the presence of the Au dopant in Ag_24_Au_1_(DMBT)_18_ strongly influenced photoluminescence, which manifested in a notable blue shift to 870 nm (Figure 2b) and strong enhancement of PL, with PLQY equal to 29.91%. Interestingly, doping with more gold atoms did not provide a further PLQY increase, but the PL of Ag_25–*x*_Au_*x*_ nanoclusters were red-shifted to 1015 nm (Figure 2b), with PLQY = 0.49%. To evaluate 2PA, the 2PEL technique was used with excitation in the NIR-II region from 1180 to 1600 nm. The σ_2_ spectra of Ag_25_ and Ag_24_Au_1_ closely matched the corresponding one-photon excitation spectra at twice the wavelength. The measured σ_2_ values for Ag_25_(DMBT)_18_ and Ag_24_Au_1_(DMBT)_18_ are on the order of 50.1 ± 22.0 GM at 1400 nm and 67.2 ± 9.9 GM at 1225 nm, respectively. Ag_25_(DMBT)_18_ and Ag_25–*x*_Au_*x*_(DMBT)_18_ exhibited the highest 2PA at shorter wavelengths within the NIR-II region, with maximum σ_2_ values of 881.3 ± 386.6 and 894.4 ± 154.9 GM for Ag_25_(DMBT)_18_ and Ag_25*x*_Au*x* (DMBT)_18_, respectively. Gold atom doping led to the enhancement of σ_2_ in the range of the lowest-energy transition band, with values increasing from 50 GM (Ag_25_) to 67 GM (Ag_24_Au_1_) and 68 GM (Ag_25–*x*_Au_*x*_) (Figure 2d–f). To evaluate the potential of application of nanoclusters in 2PM, the two-photon brightness of nanoclusters was calculated (Figure 2c). Ag_24_Au_1_ exhibited the highest σ_2_,_B_ = 20 GM, when comparing the lowest-energy transition bands (>1300 nm) of Ag_25_, Ag_24_Au_1_, and Ag_25–*x*_Au_*x*_. An Important aspect for potential application is also the fact that single Au doping resulted in increased NCs photostability. Thus, by controlling the content of metal atoms in multimetal nanoclusters one can tune their 1P and 2PEL in the NIR-I and NIR-II regions in terms of emission maxima position and PLQY. Recently, Yuan et al. also presented a single-atom doping effect on the second-order nonlinear optical properties of M_1_Ag_24_(SR)_18_ nanoclusters (M – Pd Ag/Au/Pt; SR – DMBT).^69^ Single-platinum-atom doping resulted in the largest enhancement in NIR-I photoluminescence upon 1P and 2P excitation. It also resulted in increased photostability and record-high first-order hyperpolarizability: 1001 × 10^–30^ esu at 800 nm excitation.

**Figure 2:**
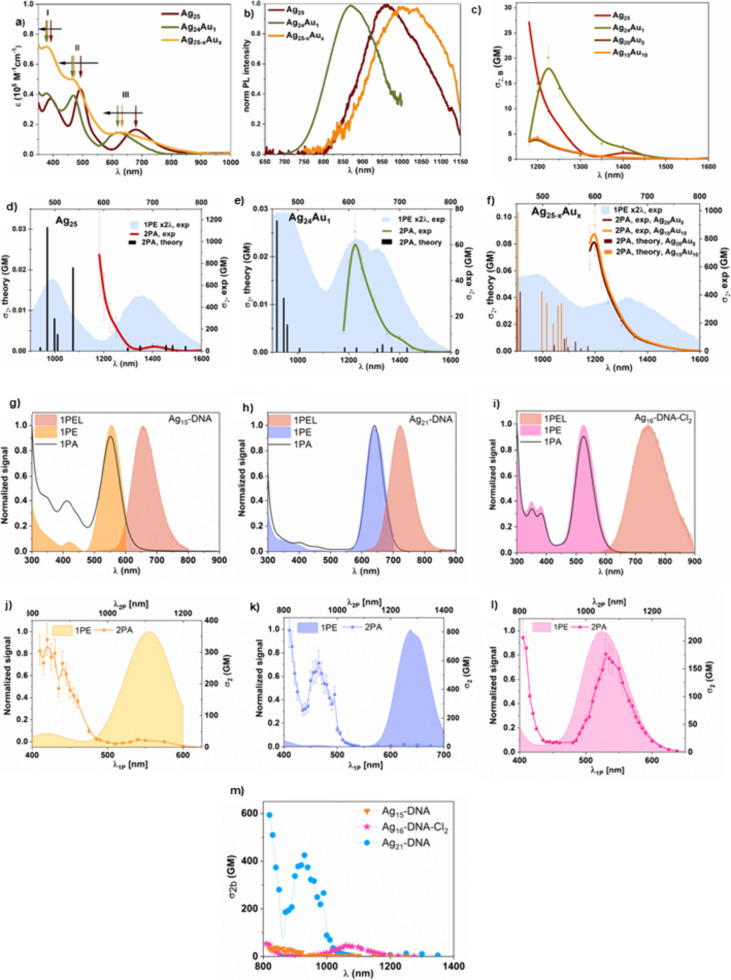
(a) 1PA spectra. (b) PL spectra and (c) σ_2,B_ of Ag_25_(DMBT)_18_ (red), Ag_24_Au_1_(DMBT)_18_ (green), and Ag_25–*x*_Au_*x*_(DMBT)_18_ (orange). (d–f) Experimental and simulated 2PA to 1PE for Ag_25_, Ag_24_Au_1_, and Ag_25–*x*_Au_*x*_. (a–f) Reproduced from ref 70. (g–i) 1PA, 1PE, and 1PEL (one-photon excited luminescence) spectra of (DNA)_2_[Ag_16_Cl_2_]^8+^, (DNA)_2_[Ag_15_]^9+^, and (DNA)_2_[Ag_16_Cl_2_]^8+^ respectively. (j–l) 2PA and 1PE of (DNA)_2_[Ag_16_Cl_2_]^8+^, (DNA)_2_[Ag_15_]^9+^, and (DNA)_2_[Ag_16_Cl_2_]^8+^ respectively. (m) Two-photon brightness of (DNA)_2_[Ag_16_Cl_2_]^8+^, (DNA)_2_[Ag_15_]^9+^, and (DNA)_2_[Ag_16_Cl_2_]^8+^. (g–m) Reproduced from ref 98.

As mentioned above, Pan et al.^90^ showed that Au_9_Ag_6_ NCs present PL at ∼800 nm and 1P, 2P, and 3P excited fluorescence sensitive to solvent polarity (acetonitrile (MeCN), ethanol (EtOH), dichloromethane (DCM), ethyl acetate (EA), dimethyl sulfoxide (DMSO), and toluene). One-photon-excited PL was the most blue-shifted in MeCN (789 nm) and the most red-shifted in EtOH (802 nm). Authors observed that in toluene and DMSO, nanoclusters exhibit one-photon excitation; in DCM, EA, and EtOH, two-photon excitation; and in MeCN, three-photon excitation between 1150 and 1450 nm. It should be noted that the reported data were limited to the tail of the PL spectrum (<700 nm). This highlights the need for future investigations to include comprehensive spectral characterization to fully assess the solvent-dependent emission properties. Selective one-photon or multiphoton excitation based on the polarity and viscosity of the surrounding media is particularly interesting in terms of bioimaging applications.

### Tuning 2P Brightness and NIR Emission Position for DNA-Templated Nanoclusters

Ag_N‑_DNAs are promising NIR emitters, and early studies on their two-photon properties were very promising in terms of efficient 2PA and water solubility. However, they were obtained for a mixture of sizes (not atomically precise Ag_N‑_DNAs), which limited the understanding of the source of the results and the correlation between chemical structure and optical properties.^99,100^ To assess the potential of atomically precise Ag_N‑_DNAs as NIR-to-NIR probes for 2PM, we measured 2PA of four representatives of Ag_N_-DNA species with far-red to NIR-I emission and varying nanocluster and ligand compositions:^101^ (DNA)_2_[Ag_15_]^9+^, (DNA)_3_[Ag_21_]^15+^, and (DNA)_2_[Ag_16_Cl_2_]^8+^. A comparison of one-photon properties like 1PA, 1PE, and 1PEL for (DNA)_2_[Ag_15_]^9+^, (DNA)_3_[Ag_21_]^15+^, and (DNA)_2_[Ag_16_Cl_2_]^8+^ is presented in Figure 2g–i, respectively. σ_2_ spectra were determined with the 2PEL technique for a wide NIR-I and NIR-II wavelength range.^98^ All Ag_N_-DNAs exhibited maximum σ_2_ values of several hundred GM at <950 nm, which correspond to one-photon transitions at <475 nm, where low intensities of 1PA are observed (Figure 2j–l). In the range of the most prominent S1 → S0 transitions, corresponding to >1000 nm in the 2P regime, (DNA)_2_[Ag_15_]^9+^ and (DNA)_3_[Ag_21_]^15+^ exhibited significantly lower σ_2_ values (Figure 2j–l). Differences in selection rules and resonant enhancements in 2PA may be the reason for these phenomena. Surprisingly, the 2PA band of (DNA)_2_[Ag_16_Cl_2_]^8+^ at longer wavelengths (∼1050 nm) had 1 order of magnitude higher σ_2_ values than the other Ag_N_-DNA species (Figure 2l). Maximum values of σ_2_ for each nanocluster excited in the NIR-I and NIR-II windows are summarized in Table 1. (DNA)_2_[Ag_16_Cl_2_]^8+^ has the highest σ_2_ in the NIR-II region: 176 ± 26 GM at 1060 nm. This cluster is distinct from others due to the presence of chloride ligands in the structure. Due to its high electronegativity, chlorine atoms alter electron density and transition dipole moments, enhancing charge-transfer effects. Computational studies showed that chlorides stabilize the electronic structure of (DNA)_2_[Ag_16_Cl_2_]^8+^ by lowering the electron density in the metal core, which shifts energy levels and affects both the HOMO (highest occupied molecular orbital) and LUMO (lowest unoccupied molecular orbital).^102^

**Table 1:** Comparison of Commercially Available Far-Red and NIR-Emitting Probes and Nanoclusters Excited in the NIR-II Window via 2PA^a^

**Probe**	**σ**_**2**_ **[GM]**	**σ**_**2,b**_ **[GM]**	**λ**_**EM**_ **[nm]**	**Excitation Window [wavelength]**
mCherry^95^	25	5.5	610	NIR-II
tdTomato^95^	108	60	581	NIR-II
Alexa Fluor 647 ^96^	133	44	671	NIR-II
Cy5 ^96^	143	40	670	NIR-II
Cy7 ^96^	200	60	779	NIR-II
Alexa Fluor 680 ^96^	203	73	704	NIR-II
ICG^97^	210	6.3	813	NIR-II
Cy5.5 ^96^	286	60	695	NIR-II
(DNA)_3_[Ag_21_]^15+^ ^98^	582	425	721	NIR-I
	17	12		NIR-II
(DNA)_2_[Ag_16_Cl_2_]^8+^ ^98^	211	54	744	NIR-I
	176	45		NIR-II
(DNA)_2_[Ag_15_]^9+^ ^98^	340	37	650	NIR-I
	25	3		NIR-II
Ag_24_Au_1_(DMBT)_18_ ^70^	67*	20	870	NIR-II
Ag_25x_Au_*x*_(DMBT)_18_ ^70^	894.4*	4.25	1015	NIR-II
Ag_25_(DMBT)_18_ ^70^	881.3*	27.1	950	NIR-II

a All data was evaluated in water solutions, besides * measured in DCM.

Finally, σ_2,B_ of each Ag_N_-DNA was determined and compared with that of commercially available probes with far-red and NIR-I emission to evaluate the potential of Ag_N_-DNAs for 2PM.^96,97^ Figure 2m presents σ_2,B_ of the Ag_N_-DNA species over the range of 810–1400 nm, which spans the NIR-I and NIR-II biological tissue transparency windows. Most notably, (DNA)_3_[Ag_21_]^15+^ has both high σ_2_ and exceptionally high PLQY compared to those of commonly used NIR- I emitting fluorophores, resulting in the highest value of 2P brightness in the NIR-I window (ca. 582 GM at 930 nm, Figure 2k). (DNA)_2_[Ag_16_Cl_2_]^8+^ exhibits high σ_2,B_ in the NIR-II window (ca. 45 GM at 1060 nm, Figure 2l). Since the number of experimentally characterized nanoclusters remains limited and the theoretical studies linking Ag_N_-DNAs structure to optical properties are still in the early stages,^103,104^ it is difficult to identify clear trends. Nevertheless, we observed that the nanocluster with the highest number of silver atoms exhibited the largest overall 2PA cross sections, while in the NIR-II excitation range, the highest σ_2_ value was found for the nanoclusters containing chlorido ligands. The advantage of Ag_N_-DNA is their water solubility, which is not obvious for organic NIR emitters effectively excited by two photons^22,105^ due to the common presence of highly hydrophobic units.

The first application of (DNA)_2_[Ag_16_Cl_2_]^8+^ in two-photon fluorescence correlation spectroscopy has already been presented.^106^ (DNA)_2_[Ag_16_Cl_2_]^8+^ was loaded into liposomes to measure cerebral blood flow rates in live mice. While these clusters have emissions in the NIR-I region (maximum of PL ∼735 nm), excitation was possible above 1000 nm due to 2PE. The authors tracked liposomes with nanoclusters inside cerebral capillaries *in vivo* and mapped flow velocities with high spatial resolution, which is highly desirable for real-time visualization of cerebrovascular system dynamics.

### Design Rules for Tuning the 2P Brightness and NIR Emission Nanoclusters

Due to the limited number of systematic studies, there is no overall guidance in tailoring high PLQY in NIR and high σ_2_ of metal nanoclusters simultaneously. Based on the literature, which addresses either emission enhancement or modulation of σ_2_, we can suggest which structural modifications lead to high two-photon brightness and emission shifted into NIR (Figure 3). The assembly of gold nanoclusters as well as the doping of silver nanoclusters with Au or Pt atoms is a strategy to increase photoluminescence^107,108^ (also in NIR-II^48,109^ ) and σ_2_.^83,110^ Gold^70^ and platinum^69^ doping into silver nanoclusters results in increased photostability and higher PLQY, with modulation of σ_2_ ^70^ and high first-order hyperpolarizabilities.^69^ For thiol-stabilized gold nanoclusters, the silver doping effect on σ_2_ depends on the cluster size.^111^ Silver doping on Au_25_ gave longer PL wavelengths,^112^ emission in NIR-II, and high σ_2_. It is also important to take into account the geometry of obtained Au_25_.^113^ There are reports on boosting PL efficiency by Cu doping on Au_25_,^53^ also in the NIR-II region.^55^ Increasing the number of metal atoms in the core increases σ_2_.^86,111,114,115^ Au_38_(PET)_24_ shows significantly stronger σ_2_ than smaller Au_25_(PET)_18_.^114^ The same trend was observed for Ag_11_, Ag_15_, and Ag_31_, capped with glutathione.^86^ Alternatively, σ_2_ values might be further tuned by cluster self-assembly, i.e., covalent linking^83^ or noncovalent interactions.^110^ However, PLQY modulation is not linearly dependent on the metal core size. Only for elongated/rod-shaped nanoclusters does the shifting of PL toward longer wavelengths occur with increasing size, entering NIR-II.^68,116,117^ Larger σ_2_ values may be also reached by ligand shell design, where aromatic, electron-donating, and core-distorting ligands were identified to enhance the 2PA transition strength. Recently, strong orbital hybridization between π-conjugated ligands and the nanocluster LUMO manifold were found to enhance 2PA.^87^

**Figure 3:**
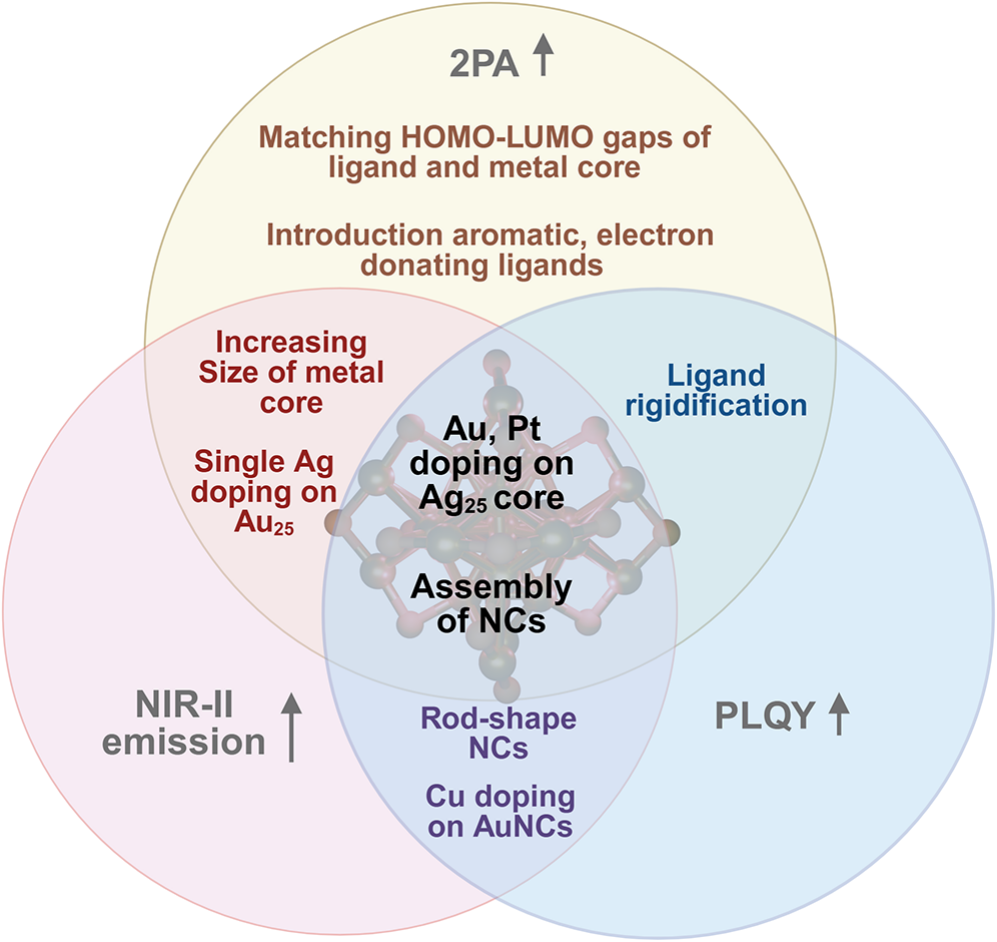
Schematic representation of approaches leading to emission in NIR-II (red), higher PLQY (blue), and σ_2_ (yellow). The overlapping area indicates approaches, which tune more than one optical property. Based on references 48, 53, 55, 68–70 ,^82^,^83^,^86^,^87^, and^107–117^ .

## Perspectives

NIR imaging offers a range of benefits in comparison to shorter wavelengths, where the NIR-II imaging window significantly improves resolution and penetration depth over NIR-I. However, accessing the emission and/or excitation wavelengths in the NIR-II region remains a major technical hurdle. To facilitate excitation above 1000 nm, multiphoton absorption is a viable alternative. Currently, the selection of fluorophores combining strong two-/three-photon absorption above 1000 nm with high photoluminescence efficiency is limited. This challenge makes noble metal nanoclusters highly promising, given their robust two- and three-photon characteristics, superior photostability, and versatile NIR-I and NIR-II emission. However, the number of *in vivo* studies using multiphoton imaging is limited, as compared to one-photon microscopy. Thus, future directions can be proposed in order to expand applications and improve our understanding of structure–property relationships in the nonlinear optical performance of nanoclusters:

### Applications

- Multiphoton microscopy and *in vivo* studies require systematic evaluation of various metrics such as imaging depth, resolution, signal-to-noise ratio, and photostability under two-photon excitation. These are not yet determined for nanoclusters. Also understanding the biological activity of NCs is still limited, as compared to that of organic compounds, and more systematic studies of NC (cyto)­toxicity, biodistribution, and accumulation in organs are needed.
- The combination of strong nonlinear absorption and high damage thresholds suggest that metal nanoclusters could serve as optical limiting agents and an effective platform for passive optical protection devices.^118–120^ The development of nanocluster-based optical limiters may open a new technological direction, which is particularly relevant in the context of advanced photonic systems and the rapidly growing defense sector in NIR-I and NIR-II.
- NCs with absorption and emission peaks between 1000 and 2000 nm^121–123^ may address the growing demand for short-wave infrared (SWIR) markers, for fields like bioimaging, civil engineering, manufacturing, the military, and many more. However, the yield and scale of syntheses have to be increased if these nanoclusters are to be used in the fabrication of commercial devices.
- Multiphoton chiral properties of Au_38_(PET)_24_,^89,124^ with circular dichroism (CD)^125^ ranging from UV to NIR as well as Au_25_ NC,^126^ were presented recently and 2P and 3P circular dichroism was proven to be stronger by 2 orders of magnitude than one-photon CD.^127,128^ The multiphoton circular dichroism of coinage metal nanoclusters may offer prospective applications in chirality detection and light modulation in a wide range of NIR wavelengths, which overcomes the drawbacks of usual CD detection in UV.

### Structure–Property Relationships in Linear and Nonlinear Optical Performance

- NLO characterization of nanoclusters has recently been developed; however, most of the data on 2PA and 3PA is restricted to single wavelengths. More systematic studies in a wide wavelength range are needed.
- A deeper understanding of NC emission in NIR is hampered by inconsistencies in NC spectra presented in the literature. As the detectors’ quantum efficiency is low for the Si detector >800 nm and InGaAs detectors >1000 nm, spectra in the 800–1000 nm range depend heavily on the equipment. Moreover, precise control of the NIR emission position is a complex problem,^67^ and further studies are needed here.
- Ag_N_-DNA PL and absorption properties are understood more deeply than for other nanoclusters due to the large-scale investigation harvesting machine learning (ML) approaches. A similar approach for monolayer-protected nanoclusters would be beneficial. However, while Ag_N_-DNAs have simple synthesis procedures but a challenging purification step, [M_*n*_(XR)_*m*_]^*q*^ often has a complex synthesis procedure, which limits the use of high-throughput procedures to produce a large number of nanoclusters in one experiment.
- The development of computational approaches like TD-DFT and ML that focus on the optical properties of NCs should be pushed toward an understanding of emission mechanisms, especially for systems with NIR and dual emission.^129^ Similar effort will be valuable in the field of theoretical description for 2PA and 3PA. Even though several approaches to enhancing σ_2_ have already been proposed (increasing the transition dipole moment, reducing the detuning energy, and introducing in-resonance effects), their realization with specific clusters still needs combined experimental and theoretical work.
